# Screening for diabetic peripheral neuropathy at community pharmacies in Slovakia

**DOI:** 10.3325/cmj.2024.65.85

**Published:** 2024-04

**Authors:** Zuzana Pagáčová, Milan Grofik, Tomáš Fazekaš, Viera Žufková, Daniela Mináriková

**Affiliations:** 1 Department of Organisation and Management of Pharmacy, Faculty of Pharmacy, Comenius University, Bratislava, Slovakia; 2 Department of Neurology, Jessenius Faculty of Medicine in Martin, Comenius University Bratislava, Bratislava, Slovakia; 3 Department of Physical Chemistry of Drugs, Faculty of Pharmacy, Comenius University Bratislava, Bratislava, Slovakia; 4 Department of Languages, Faculty of Pharmacy, Comenius University Bratislava, Bratislava, Slovakia; 5 Department of Organisation and Management of Pharmacy, Faculty of Pharmacy, Comenius University Bratislava, Bratislava, Slovakia

## Abstract

**Aim:**

To identify diabetic patients with a potential risk of developing diabetic peripheral neuropathy (DPN) in community pharmacies in Slovakia using a modified Michigan Neuropathy Screening Instrument questionnaire (MNSIq-12).

**Methods:**

This cross-sectional study enrolled 703 patients with type 1 and type 2 diabetes mellitus who had not been diagnosed with DPN. The study took place in selected community pharmacies across Slovakia in October 2019. The MNSIq-12 was administered by pharmacy students, and a Michigan score <1.5 was considered risky. The groups divided based on the Michigan score were compared in terms of duration of diabetes, age, body mass index (BMI), sex, weekly physical activity, level of education, and smoking.

**Results:**

The risk of developing DPN was detected in 6.6% of respondents with type 1 diabetes and 13.4% with type 2 diabetes. Patients with both types of diabetes (38.2%; 67.0%) reported fatigue and heaviness in the legs as the most common clinical symptoms that may indicate the development of DPN. Those with a Michigan score <1.5 were older (*P* < 0.0001), had a higher BMI (*P* < 0.0001), a lower level of education (*P* = 0.0020), and were less physically active (*P* < 0.0001).

**Conclusion:**

Approximately one-eighth of patients with diabetes who visited community pharmacies were potentially at risk for developing DPN. The modified MNSIq-12 was shown to be a simple, time-effective, and non-invasive indicative screening tool that can be applied in the environment of community pharmacies.

According to the International Diabetes Federation estimates, more than 425 million people worldwide have diabetes, which makes it the largest global epidemic (1). In Slovakia in 2021, 355 000 patients were treated for diabetes and 21 000 new patients were diagnosed (2).

Diabetic peripheral neuropathy (DPN), in its painful and non-painful form, is one of the most common chronic complications in patients with diabetes mellitus. It is a neurodegenerative disorder of the peripheral nervous system, preferentially affecting sensory axons, autonomic axons, and to a lesser extent motor axons. It is manifested as tingling or a burning sensation in the toes, feet, and calves, as well as the formation of defects that can lead to the amputation of a limb (3,4). The pathophysiological mechanisms of DPN differ between type 1 and type 2 diabetes. Correlations between nerve damage extent and serological parameters indicate that nerve lesions in patients with type 1 diabetes are predominantly associated with poor glycemic control and loss of nerve conduction, while in patients with type 2 diabetes they are associated with changes in lipid metabolism (5).

Several screening methods are available to assess the structure and function of the peripheral nervous system, each with advantages and disadvantages (3,6). Clinical tests are a fundamental screening tool because they are simple, economical, and implementable in everyday conditions (community pharmacy, ambulance, at home) (7). The Michigan Neuropathy Screening Instrument (MNSI) is the most commonly used tool to assess distal symmetrical DPN, with confirmed reliability and validity (8-10). It includes two separate evaluations: a 15-item questionnaire that the diabetic patient can fill out individually and a medical specialist´s examination of the lower limbs that assesses vibration sensitivity, ankle reflexes, and visually detectable changes typical of neuropathy (ulcerations, fissures, foot deformities, and dry skin). Points are assigned based on the presence or absence of disturbances in vibration sensitivity, ankle reflex, and changes observed in aspect (7,11).

To minimize the long-term risk of chronic complications, patients with diabetes mellitus need to adhere to the prescribed treatment and perform regular self-monitoring, even with the help of a pharmacist (12). Globally, the role of pharmacists is expanding from that of dispensing medications to performing basic biochemical measurements and providing counseling services. Pharmacists also play a key role in chronic disease management (13). The involvement of pharmacists in the management of diabetes mellitus reduces the costs of health care and, thus, increases the efficiency of the provided care (12-15). However, studies looking at the involvement of pharmacists in the prevention of complications of diabetes mellitus in the environment of community pharmacies are sparse (16).

This study aimed to use the modified Michigan Neuropathy Screening Instrument questionnaire (MNSIq-12), a simple 12-item indicative screening tool, in the conditions of community pharmacies in Slovakia to capture the potential risk of developing DPN among diabetic patients.

## Patients and methods

This cross-sectional study was carried out in 65 community pharmacies across Slovakia for two weeks in October 2019. The study was approved by the Ethics Committee for Biomedical Research of the Faculty of Pharmacy of Comenius University Bratislava (04/2019).

### Patients

Diabetic patients were addressed in community pharmacies by 73 students of the 4th year of the master's study program at the Faculty of Pharmacy of the Comenius University Bratislava, who were at that time completing their compulsory pharmacy practice. The place of compulsory practice was chosen by the students. Before starting the practice, students participated in the educational project Advanced Training in Pharmacy Care organized by the Faculty of Pharmacy, which dealt with the diagnosis, treatment, and complications of diabetes mellitus. Their role as interviewers in gathering data did not require special training, but their participation in the educational project allowed us to specifically entrust them with data collection. During the practice under the guidance of a pharmacist, the students provided pharmaceutical care for patients, therefore their involvement in the study reflected the real conditions in the community pharmacy. The inclusion criteria were confirmed diabetes mellitus type 1 or type 2 and age ≥18 years. The exclusion criteria were already diagnosed DPN and the use of drugs containing α-lipoic acid (thioctic acid), which is used to treat sensitivity disorders and nerve damage caused by diabetes mellitus. A total of 703 patients who met the study's criteria were divided into two groups: patients with type 1 diabetes (DM1T) and patients with type 2 diabetes (DM2T) for further evaluation.

### Methods

A modified version of MNSI questionnaire - MNSIq-12 was chosen as an indicative screening tool (17). We decided to use this indicative screening tool because of its simplicity and usability in the conditions of community pharmacies. The questionnaire consisted of 12 simple questions with “yes” or “no” answers. The patients were given one point if they answered “yes” to questions 1-6, 9-12 and “no” to questions 7 and 8 due to the opposite polarity of these items. The Michigan score was calculated by adding up the points for all questions. The Michigan score of <1.5 was considered as a risk for developing DPN. Patients with an increased risk of DPN were advised to visit a diabetologist for further, more complex examinations due to the subjectivity of the data provided. The participants' demographic characteristics were determined with appropriately designed questions by the authors. The body mass index (BMI) was calculated in the standard way from body weight and height (Centers for Disease Control and Prevention guidelines), provided by the participants. Afterwards, the participants were divided into groups according to BMI. The engagement in physical activity for 30 minutes per day was self-reported.

### Statistical analysis

The normality of distribution was tested with a Shapiro-Wilk test. Descriptive statistics were employed to summarize the data, while inferential statistics (ANOVA, *t* test) were used to examine the relationship between the assessment variables. Continuous data are presented as mean, standard deviation, coefficient of variation, minimum, and maximum. Categorical variables are expressed as absolute or relative frequencies. *P* < 0.05 was considered statistically significant. The data analysis was performed with SAS Education Analytical Suite for Microsoft Windows, version 9.3 (SAS Institute Inc., Cary, NC, USA).

## Results

The study enrolled 136 patients with DM1T and 567 patients with DM2T. The average age of DM1T patients was 35.99 ± 17.83 years, and the average age of DM2T patients was 61.53 ± 11.79 years. Patients in the DM1T group were treated for diabetes considerably longer (14.78 ± 10.39 years), had a higher education (42.6% to 29.6%), and a lower average BMI (23.97 ± 3.94 kg/m^2^) than patients in the DM2T group (Table 1).

**Table 1:** Characteristics of patients with diabetes mellitus involved in the study (N = 703)

	No. (%) of participants with	
Characteristics	diabetes mellitus type 1	diabetes mellitus type 2
Proportions (%)	136 (19.35)	567 (80.65)
Age (years)	35.99 ± 17.83	61.53 ± 11.79
Sex		
male	57 (16.9)	280 (83.1)
female	79 (21.6)	287 (78.4)
Duration of treatment (years), mean ± standard deviation	14.78 ± 10.39	10.19 ± 9.41
Body mass index (BMI) (kg/m^2^); mean ± standard deviation	23.97 ± 3.94	29.43 ± 4.72
BMI classification		
underweight (<18.5 kg/m^2^)	7 (5.1)	0 (0)
normal weight (18.5-24.9 kg/m^2^)	76 (55.9)	66 (11.6)
overweight (25-29.9 kg/m^2^)	40 (29.4)	270 (47.6)
obesity class I (30-34.9 kg/m^2^)	11 (8.1)	174 (30.7)
obesity class II (35-39.9 kg/m^2^)	2 (1.5)	38 (6.7)
obesity class III (≥40 kg/m^2^)	0 (0)	19 (3.4)
Education		
primary	16 (11.8)	51 (9.0)
secondary	62 (45.6)	348 (61.4)
university	58 (42.6)	168 (29.6)
Smoking		
yes	35 (25.7)	157 (27.7)
no	101 (74.3)	410 (72.3)

Patients in both groups reported fatigue and heaviness in the legs as the most frequent clinical symptom predicting the development of DPN (38.2% for DM1T group; 67.0% for DM2T group). The least frequent symptom was the inability to distinguish between hot and cold water in the DM1T group (2.9%) and amputation (including partial) in the DM2T group (1.9%). The incidence of all clinical symptoms (Table 2) was higher in the DM2T group, but the difference reached significance only for several of them. Patients from the DM2T group more frequently reported fatigue and heaviness in legs (*P* < 0.0001), prickling feeling in legs or feet (*P* < 0.0001), dry skin on feet with cracks (*P* < 0.0001), burning pain in legs and/or feet (*P* < 0.0001), decreased sensitivity in feet or hands (*P* < 0.0001), worsened symptoms at night (*P* = 0.0137), feet too sensitive to touch (*P* = 0.0243), and painful sensation of blanket touching the skin (*P* = 0.0350). Other clinical symptoms did not show a significant difference.

**Table 2:** Clinical symptoms according to Michigan Neuropathy Screening Instrument questionnaire in patients with diabetes mellitus (N = 703)

	No. (%) of respondents with		
Clinical symptoms	diabetes mellitus type 1 (n = 136)	diabetes mellitus type 2 (n = 567)	P
Fatigue and heaviness in legs	52 (38.2)	380 (67.0)	<0.0001
Prickling feelings in legs or feet	43 (31.6)	289 (51.0)	<0.0001
Dry skin on feet with cracks	36 (26.5)	286 (50.4)	<0.0001
Burning pain in legs and/or feet	36 (26.5)	279 (49.2)	<0.0001
Feeling of decreased sensitivity in feet or hands	18 (13.2)	192 (33.9)	<0.0001
Worsened symptoms at night	22 (16.2)	149 (26.3)	0.0137
Inability to determine the location of pain	27 (19.9)	126 (22.2)	0.5476
Feet too sensitive to touch	15 (11.0)	109 (19.2)	0.0243
The doctor said I have diabetic neuropathy	9 (6.6)	58 (10.2)	0.1977
Painful sensation of blanket touching the skin	5 (3.7)	52 (9.2)	0.0350
Inability to distinguish between hot and cold water	4 (2.9)	32 (5.6)	0.1991
Amputation (including partial)	5 (3.7)	11 (1.9)	0.2227

The average Michigan score for the DM1T group was 1.83 ± 0.21; for the DM2T group, it was 1.71 ± 0.21. Eighty-five of 703 patients achieved a Michigan score of <1.5 (9 in the DM1T group and 76 in the DM2T group). The relative frequency of occurrence and development of DPN was significantly higher (*P* = 0.0293) in the DM2T group (Table 3).

**Table 3:** The average Michigan score in the diabetes mellitus type 1 (DM1T) and diabetes mellitus type 2 (DM2T) group (N = 703)

	DM1T (n = 136)	DM2T (n = 567)	P
Mean ± standard devaition	1.83 ± 0.21	1.71 ± 0.21	
Michigan score, n (%)			
<1.5	9 (6.6)	76 (13.4)	0.0293
>1.5	127 (93.4)	491 (86.6)	

Patients with a Michigan score <1.5 had longer duration of diabetes (17.11 ± 12.75 years vs 10.25 ± 8.96 years; *P* < 0.0001), were older (65.33 ± 13.64 years vs 55.39 ± 16.59 years; *P* < 0.0001), had a higher BMI (30.30 ± 5.83 kg/m^2^ vs 28.13 ± 4.87 kg/m^2^; *P* = 0.0002), less frequently had higher education (18.8% vs 34%; *P* = 0.0020), and did not meet the recommended level of physical activity (30 min per day) (14.1% vs 39.8%; *P* < 0.0001) than patients with a Michigan score >1.5. The groups did not significantly differ in sex or smoking status (Table 4).

**Table 4:** Respondents’ (N = 703) characteristics according to the acquired Michigan score (MS)

Variable	MS<1.5 (n = 85); mean ± standard deviation	MS>1.5 (n = 618); mean ± standard deviation	P
Duration of diabetes mellitus (years)	17.11 ± 12.75	10.25 ± 8.96	<0.0001
Age (years)	65.33 ± 13.64	55.39 ± 16.59	<0.0001
Body mass index (kg/m^2^)	30.30 ± 5.83	28.13 ± 4.87	0.0002
Sex, n (%)			0.8627
male	40 (41.7)	297 (48.1)	
female	45 (52.9)	321 (51.9)	
Weekly physical activity (30 min/d), n (%)	12 (14.1)	246 (39.8)	<0.0001
Level of education, n (%)*			0.0020
lower education	69 (81.2)	408 (66.0)	
higher education	16 (18.8)	210 (34.0)	
Smoking	25 (29.4)	167 (27.0)	0.6430

*lower education = primary and secondary; higher education = university.

## Discussion

The majority of studies aimed at pharmacist-led interventions in the management of diabetes are mainly focused on disease screening, education, and contribution of pharmacists to improving patients' self-management. This study is one of a few and the first in Slovakia to investigate the use of the Michigan score to identify diabetic patients potentially at risk of developing DPN in the setting of community pharmacies. In this study, there was a notable proportion of patients potentially at risk of DPN who should be examined by a specialist to confirm the diagnosis of DPN. Accordingly, the study shows that pharmacist-led interventions using a simple, reliable, and non-invasive tool (16,18,19) can play a valuable role in screening and counseling diabetic patients about DPN and its risk factors. Since DPN affects a varying number of patients with diabetes (from 10% to 90%) (20,21), early diagnosis appears to be the best strategy in the care of the diabetic patient. Timely recognition and appropriate interventions hold promise in slowing down or even reversing the early stage of nerve fiber damage in people with diabetes (22,23). Currently, DPN screening by community pharmacists is not routinely performed in Slovakia. Nevertheless, incorporating this tool in the prevention of complications associated with diabetes within community pharmacies is worthy of consideration. We were able to identify a considerable number of diabetic patients who may be at risk for DPN. By evaluating the answers to the MNSIq-12, we concluded that several clinical symptoms showed importance. The most common subjective difficulty in our sample was the feeling of fatigue and heaviness in the legs. However, polyneuropathy can have a multitude of etiologies, and its clinical manifestations can vary from asymptomatic to painful neuropathic symptoms.

Approximately one-eighth of diabetic participants in our study achieved a Michigan score <1.5, which indicates an increased risk of developing DPN. Achieving the Michigan score <1.5 was strongly associated with duration of diabetes treatment, age, BMI, physical activity levels, and education. Our observations are consistent with the findings of several recent studies (24-28).

Khawaja et al and Popescu et al reported an association between age, duration of diabetes, and the risk of developing neuropathy (25,29). Our study revealed that the risk of achieving a Michigan score <1.5 was higher among patients with a longer duration of diabetes mellitus and older age. Therefore, our findings emphasize the need to particularly focus on screening for DPN in elderly patients and those with longstanding diabetes. Moreover, no sex differences were observed.

In line with other reports, our findings revealed that patients with a higher BMI (>30 kg/m^2^) were more likely to achieve a Michigan score <1.5 (30,31). Metabolic syndrome and obesity are associated with an increased risk of developing DPN (25,30,32). At the same time, the question arises whether obesity alone is sufficient to trigger the mechanisms leading to the development of neuropathy in diabetic patients. Further research is needed to clarify this issue.

Physical activity is essential in preventing many chronic diseases, including diabetes and its complications (33). Dixit et al (34) showed a positive effect of regular physical activity on the functionality of the peripheral nervous system, which is affected in DPN. However, there is a lack of clear evidence for the effectiveness of physical activity and dietary interventions from large, randomized control trials (35). In our research, we observed a relevant relationship between engaging in regular weekly physical activity for 30 minutes and reaching a Michigan score ≥1.5.

Similar to the findings of Azmiardi et al (24), we confirmed a notable relationship between the level of education and the risk of developing DPN. Among our participants, those with a Michigan score of <1.5 were less likely to have higher education and thus were more at risk for developing DPN. This prompts us to consider to what extent improving the health literacy of diabetic patients could contribute to reducing health disparities related to education level. Good health literacy skills are essential for preserving and improving health (36).

We did not observe a higher probability of smoking among patients with a Michigan score <1.5 compared with patients who achieved a Michigan score ≥1.5. However, smoking is known to be associated with oxidative stress, systemic inflammation, and endothelial dysfunction. This factor increases the risk of nerve damage and compromises the integrity of the skin and the ability of diabetic patients to heal wounds (31,37).

This study emphasizes the importance of integrating pharmacist-led interventions for early detection of DPN and enhancing the implementation of health education mostly among the elderly, obese, and physically inactive patients with long-standing diabetes. Identifying variables connected with a Michigan score <1.5, and consequently a heightened risk of developing DPN, is crucial in the treatment of diabetes and in the prevention of complications associated with this disease.

This study has several limitations. The duration of physical activity and BMI were determined by an indirect method, which could lead to less accurate data. Another potential limitation of the study could be that alcohol consumption was not included among variables probably linked to an increased risk of developing DPN.

In conclusion, while screening for DPN is strongly recommended in clinical practice, it is frequently overlooked in the management of diabetic patients. Our research confirms that MNSIq-12, as a simple, time-effective, and non-invasive indicative screening tool, may be applied in community pharmacies to identify patients with DPN.
